# Changing from analog to digital images: Does it affect the accuracy of alignment measurements of the lower extremity?

**DOI:** 10.3109/17453674.2011.570670

**Published:** 2011-07-08

**Authors:** Martina Lohman, Kaj Tallroth, Jyrki A Kettunen, Ville Remes

**Affiliations:** ^1^Helsinki University Central Hospital; ^2^ORTON Orthopaedic Hospital; ^3^ORTON Research Institute, ORTON Foundation; ^4^Arcada University of Applied Sciences, Helsinki, Finland

## Abstract

**Background and purpose:**

Medical imaging has changed from analog films to digital media. We examined and compared the accuracy of orthopedic measurements using different media.

**Methods:**

Before knee arthroplasty, full-length standing radiographs of 52 legs were obtained. The mechanical axis (MA), tibio-femoral angle (TFA), and femur angle (FA) were measured and analyzed twice, by 2 radiologists, using (1) true-size films, (2) short films, (3) a digital high-resolution workstation, and (4) a web-based personal computer. The agreement between the 4 media was evaluated using the Bland-Altman method (limits of agreement) using the true-size films as a reference standard.

**Results:**

The mean differences in measurements between the traditional true-size films and the 3 other methods were small: for MA –0.20 to 0.07 degrees, and for TFA –0.02 to 0.18 degrees. Also, the limits of agreement between the traditional true-size films and the three other methods were small.

**Interpretation:**

The agreement of the alignment measurements across the 4 different media was good. Orthopedic angles can be measured as accurately from analog films as from digital screens, regardless of film or monitor size.

During the last 15 years a gradual change from traditional, analog film radiographs to digital imaging has occurred. This has implemented a change not only in the way images are obtained, but also in the way images are archived and, above all, evaluated. The image transition time in daily clinical practice has decreased (May et al. 2000). Traditional film evaluation has changed to image reading on monitor screens; this change affects not only radiologists, but also orthopedic surgeons. In addition to the change in the visual analysis of images, there has also been a change in how measurements are performed. Light boxes, rulers, and grease pencils have been exchanged for computers using graphics software.

In knee surgery, it is common to assess the alignment of the whole lower extremity with a hip-to-ankle radiograph from which the mechanical axis (MA), the tibio-femoral angle (TFA), and femur angle (FA) are measured. Excellent intra- and interobserver reproducibility of analog MA measurements has been reported in several studies (Henderson and Kemp 1991, Sharma et al. 2001, Rauh et al. 2007, Gordon et al. 2009). In osteoarthritis, the tibio-femoral joint space narrowing is often unsymmetrical, leading to angular deformity of the lower extremity, more commonly of varus type. In order to be able to restore the alignment, knowledge of the preoperative malalignment is crucial. A malalignment of the knee prosthesis not only increases the likelihood of postoperative malfunction, but also affects the lifespan of the prosthesis (Sorrells et al. 2007).

We investigated the accuracy of alignment measurements performed using 4 techniques: analog true-size films, films of reduced size, high-resolution workstations, and web-based personal computers (PCs). Before that, we assessed the intra- and interobserver reliability of these 4 techniques, separately for each technique.

## Patients and methods

### Study material

This was a prospectively planned study of 52 whole-leg radiographs from 40 consecutive patients (mean age 70 (39–86), 28 women) undergoing total knee arthroplasty in our hospital. The indication for knee surgery was degenerative osteoarthritis (47 knees), rheumatoid arthritis (1 knee), posttraumatic sequelae (1 knee), spastic paraplegia (1 knee), and hemophilia (1 knee). Both knees were operated in 12 patients. The alignment of the preoperative knees was as follows: deviation of MA 1–24^o^ varus (mean 9.8^o^), TFA 0–28^o^ varus (mean 4.4^o^), and FA 3–10^o^ (mean 6.5^o^).

### Imaging technique

The digital radiographs of the lower extremity were obtained as part of the patient's preoperative planning routine. The patients stood barefoot on both legs with the medial aspects of the feet parallel. A standardized technique was used: the X-ray beam was centered on the affected knee, and the focus-imaging plate distance was 2.1 m. If both knees were scheduled for surgery, each leg was radiographed separately.

The digital pictures were retrieved from our PACS (picture-archiving and communications system) and printed both on a long whole-leg film of 35 × 130 cm (true size) and on a short film of 35 × 43 cm (41% of the true size), and also analyzed on a high-resolution monitor of a diagnostic workstation (resolution 1,600 × 1,200, active screen 21.3 × 17.0 inches, type Coronis 2MP; Barco N.V., Kortrijk, Belgium) and from a web-based PC monitor meant for clinicians (resolution 1,280 × 1,024, active matrix screen 14.9 × 12.0 inches, type HP dc7600 SFF Cel D-336 40G 256 M 4PC; Hewlett Packard, Houston, TX).

### Image analysis

Each of the 52 leg images was analyzed using the 4 media. The evaluations for this intra- and interobserver reliability study were carried out by 2 senior musculoskeletal radiologists (KT and ML) independently of each other who were blinded regarding previous readings. Both observers evaluated all the images from the films and monitor screens twice on different occasions. A goniometer was used for the angle measurements on the films, and for the angle measurements on the monitor screens, measurement tools of the workstation and PC were used.

**Figure 1.**
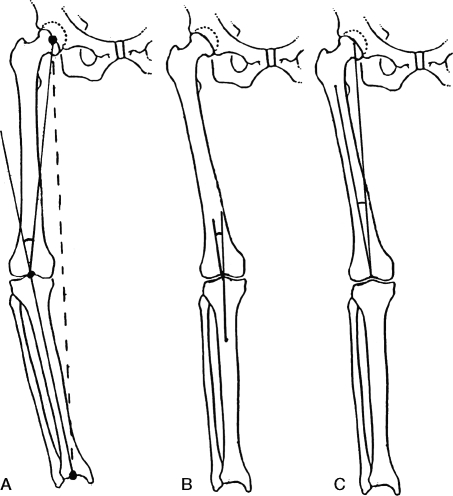
Schematic pictures showing the methods for radiographic assessment of mechanical axis (A), tibiofemoral angle (B), and femoral angle (C).

The MA angle was calculated according to Hagstedt et al. (1980). For this calculation, the center of the femoral head was defined by using Mose (1980) circles, the midpoint of the knee being defined by the center of the femoral condyles at the level of the top of the intercondylar notch, and the midpoint of the ankle being defined by the center of the superior facet of the talus. The TFA is formed by the intersection of a line through the femoral midcondylar point and the center of the distal femoral shaft with that of a line through the proximal shaft of the tibia (Moreland et al. 1987). The FA was defined as the angle between a line (the femoral mechanical axis) through the center of the femoral head and the midpoint of the knee and a line (the femoral shaft axis) from this midpoint to the middle of the proximal femoral diaphysis at the level of the lesser trochanter (Moreland et al. 1987) (Figure 1).

The MA, TFA, and the FA were all evaluated from the same radiographs, shown both on the 2 printed films of different size and on the 2 different types of monitors. Due to lack of a gold standard, we were forced to create reference values for MA and TFA based on the traditional long films. These reference values consisted of the mean value of the 4 readings, i.e. 2 readings by each of the 2 raters. Similarly, we calculated the values for the measurements of MA and TFA for each patient in the 3 new media. In the comparison of the 4 media, FA was excluded as it showed a slightly weaker reliability in some of the comparisons (see Results).

### Statistics

**Figure 2.**
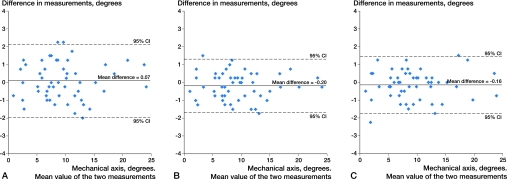
Bland-Altman plots showing differences from mean for mechanical axis measurements in 52 legs, comparing true-size films to short films (A), radiological workstation (B), and PC (C).

**Figure 3.**
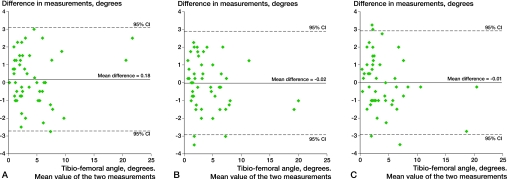
Bland-Altman plots showing differences from mean for tibio-femoral angle measurements in 52 legs, comparing true-size films to short films (A), radiological workstation (B), and PC (C).

The reliability of the measurements was evaluated by calculating the intraclass correlation coefficient (ICC) (Shrout and Fleiss, 1979). Intraobserver repeatability was calculated with a 1-way random model, and all the other calculations were done with a 2-way random model. For the interobserver differences, the first readings of the raters were used. To analyze the agreement between the traditional long films and the three new media, the Bland-Altman method was used. In this method, the differences in measurements between the 2 methods are plotted against each individual's mean of the measurements for the 2 methods. In Figures 2 and 3, the solid line represents the mean difference (bias), and the dashed lines represent the 95% confidence intervals (CIs) for the mean difference. The analyses were performed using SPSS version 17.0.

## Results

The ICC was between 0.90 and 0.99 for measurements of MA and TFA, indicating excellent intra- and interobserver reliability, but for FA it ranged between 0.50 and 0.88.

When using the traditional true-size films as a reference standard and comparing the 3 other methods to this, the mean differences and the limits of agreement between the methods were small: for MA –0.20 to 0.07 degrees, and for TFA –0.02 to 0.18 degrees (see Figures 2 and 3). The maximum difference of the MA measurements in our subjects in the comparison of the media was always less than 3 degrees; for the TFA, in 4 cases the maximal individual measurement error exceeded 3 degrees per comparison.	

## Discussion

We found excellent intra- and interobserver reliability for both the MA and TFA measurements from all 4 media, i.e. true-size films, reduced-size films, diagnostic workstation, and PC. Our comparison of the 4 methods showed excellent agreement for the MA and TFA measurements, and it was only slightly weaker for FA. We conclude that all 4 methods of lower limb measurement are highly reliable for assessment of knee malalignment.

High-quality radiographs and reliable measurements are essential for preoperative planning of knee arthroplasty. The objective is to position the implants in order to restore the mechanical axis of the limb. Thus, full-length weight-bearing radiography is required for evaluation of preoperative malalignment and to estimate the correction needed. FA, i.e. the angle between the femoral mechanical axis and femoral shaft axis, is important for the accurate insertion of a distal femoral intramedullary cutting guide for placement of the femoral component perpendicular to the mechanical axis.

The intra- and interobserver variation of orthopedic measurements performed on traditional radiographs has proven not only to be sufficient, but good when performed by experienced radiologists (Moreland et al. 1987, Carman et al. 1990, Ylikoski and Tallroth 1990, Henderson and Kemp 1991, Oswald et al. 1993, Ritter et al. 1994, Sharma et al. 2001, Feldman et al. 2007, Rauh et al. 2007). In recent years, analog films have been increasingly replaced by digital images. This has changed the daily work routine for technicians, clinicians, and radiologists. Rulers and protractors have been replaced by digital measuring tools.

Digital image analysis and viewing can be performed from dedicated radiological workstations or from PCs. Dedicated high-resolution workstations are more commonly used by radiologists, while clinicians mainly view and analyze their images using PCs. Dedicated workstations are expensive, and usually they have better image resolution and a better supply of functions to facilitate the analysis. The PC programs are usually web-based, and the image quality is largely dependent on the combination of PC and screen used. However, in 2 studies, PC-based image viewing was deemed useful (Parasyn et al. 1998, Prakash et al. 2001), although these studies did not compare the accuracy of PC-based image analysis to that performed on dedicated diagnostic workstations. In a phantom study, the image analysis performed from a radiological workstation was found to be superior to that done using a PC, even when image processing was performed (Ricke et al. 2000).

Digitally performed measurements have been considered more reliable than analog measurements for hallux valgus measurements (Farber et al. 2005) and relatively equal for measurements of idiopathic scoliosis (Carman et al. 1990, Shea et al. 1998, Zmurko et al. 2003, Kuklo et al. 2006) and for measurement of the mechanical axis of the lower limb (Takahashi et al. 2004, Sailer et al. 2005). Even when performed by non-medical staff, computerized measurements of the tibio-femoral alignment have been considered reliable (Prakash et al. 2001). In the study by Sled et al. (2011), the accuracy of measurements of lower limb alignment performed on analog films and digital images by 14 trained but not further characterized raters (7 for each method) was compared, and found to be highly reliable. In a study by Sailer et al. 2005) on axial alignment of the lower extremity, analog and digital radiographs obtained on separate occasions were found to be similar. Goker and Block (2007) found reasonable agreement when evaluating MA measurements performed manually and digitally in 28 knees with osteoarthritis. Also, Specogna et al. (2004) found minimal measurement differences between manual and digital measurements of the MA in a study in which originally analog images were later digitized.

All our radiographs were taken by experienced radiographic technologists using standardized positioning and a standardized technique. We consider that the strength of our study design is that the images analyzed from 4 media, both analog and digital, originated from the same original hip-to-ankle radiographs. This made it possible to avoid misinterpretations and miscalculations due to different picture positioning, exposure, and limb flexion and rotation, which might easily influence the measurement results. Secondly, before the study both radiologists had agreed on the definition of the landmarks, and they used the same goniometer for the analog measurements and the same digital software in the digital analyses. These factors certainly contributed to the small variation between the measurements.

We found no systematic error in any of the measurements. This can partly be explained by the fact that both of the raters were experienced musculoskeletal radiologists. The minor differences in angle measurements when comparing the media are of no clinical significance. The severity of preoperative malalignment of the extremity had no effect on the measurement error.

FA is defined by 3 landmarks, 2 of which are also used for MA measurements. Consequently, the variation in FA measurements is due to the identification of the third landmark, i.e. the middle of the proximal femur at the level of the lesser trochanter. This is surprising and difficult to explain, as the two experienced film readers had agreed on the precise definition of all landmarks. However, it should be remembered that although the interclass correlation was weak for FA, the mean difference in the angle measurements was less than one degree.

In conclusion, orthopedic angle measurements for the lower extremity aligment showed a high degree of consistency and reproducibility. FA, used in the execution of the femur cut at arthroplasty, showed a moderate ICC—but in both the intra- and interobserver readings, the mean difference was less than one degree. The measurements performed on a computer screen are as accurate as those performed on traditional true-size films when evaluating alignment changes and planning surgery.
